# Retest reliability of individual alpha ERD topography assessed by human electroencephalography

**DOI:** 10.1371/journal.pone.0187244

**Published:** 2017-10-31

**Authors:** Manuel Vázquez-Marrufo, Alejandro Galvao-Carmona, María Luisa Benítez Lugo, Juan Luis Ruíz-Peña, Mónica Borges Guerra, Guillermo Izquierdo Ayuso

**Affiliations:** 1 Experimental Psychology Department, Faculty of Psychology, Universidad de Sevilla, Sevilla, Spain; 2 Department of Psychology, Universidad Loyola Andalucía, Sevilla, Spain; 3 Physiotherapy Department, Faculty of Nursing, Physiotherapy and Chiropody, Universidad de Sevilla, Sevilla, Spain; 4 Multiple Sclerosis Unit, Virgen Macarena Hospital, Sevilla, Spain; Universita degli Studi di Roma La Sapienza, ITALY

## Abstract

**Background:**

Despite the immense literature related to diverse human electroencephalographic (EEG) parameters, very few studies have focused on the reliability of these measures. Some of the most studied components (i.e., P3 or MMN) have received more attention regarding the stability of their main parameters, such as latency, amplitude or topography. However, spectral modulations have not been as extensively evaluated considering that different analysis methods are available.

The main aim of the present study is to assess the reliability of the latency, amplitude and topography of event-related desynchronization (ERD) for the alpha band (10–14 Hz) observed in a cognitive task (visual oddball). Topography reliability was analysed at different levels (for the group, within-subjects individually and between-subjects individually).

**Results:**

The latency for alpha ERD showed stable behaviour between two sessions, and the amplitude exhibited an increment (more negative) in the second session.

Alpha ERD topography exhibited a high correlation score between sessions at the group level (r = 0.903, p<0.001). The mean value for within-subject correlations was 0.750 (with a range from 0.391 to 0.954). Regarding between-subject topography comparisons, some subjects showed a highly specific topography, whereas other subjects showed topographies that were more similar to those of other subjects.

**Conclusion:**

ERD was mainly stable between the two sessions with the exception of amplitude, which exhibited an increment in the second session. Topography exhibits excellent reliability at the group level; however, it exhibits highly heterogeneous behaviour at the individual level. Considering that the P3 was previously evaluated for this group of subjects, a direct comparison of the correlation scores was possible, and it showed that the ERD component is less reliable in individual topography than in the ERP component (P3).

## Introduction

Neural activity may be monitored through electroencephalographic activity (EEG), particularly in two different domains: event-related potentials (time) and frequency analysis (spectral). For the latter option, different methods may be employed to measure specific bands; for example, Fast Fourier transformation, wavelets, or event-related desynchronization (ERD) or synchronization (ERS) enable the observation of specific modulations in the EEG frequency bands [1]. An advantage from time-frequency techniques is the possibility to calculate modulations in the spectral domain in a millisecond scale. To accomplish this, different approaches have been used to include the variable “time” in the calculation. In the case of ERD, a process of filtering, rectifying or square and averaging the EEG signal permits the determination of the changes in spectral bands of EEG related to diverse processes [2].

Since the last century, many studies with ERD have been conducted to analyse diverse processes (sensorial, cognitive and motor) in the brain. The main efforts were initially oriented to motor activity or sensory stimulation [3, 4]. A more cognitive approach subsequently started to appear in the literature with different paradigms. Van Winsum et al. [5] related the ERD modulation to the attention demands of a task and the surprise value of stimuli. Other studies have also identified modulations related to the attentional status in human subjects [6, 7]. In the case of memory paradigms, different studies have shown that ERD is related to the retrieval and comparing processes, whereas ERS is more related to auditory encoding [8, 9, 10]. In more recent studies, ERD has been related to affective valence processing for emotional stimuli [11], minimally conscious state [12] or neurofeedback [13].

As for ERPs and spectral analysis with other techniques, one challenge for ERD measures is their test-retest reliability in longitudinal studies. In the particular case of ERD and alpha band, the results have been heterogeneous between studies. Burgess and Gruzelier [14], by measuring the alpha power in the baseline period, identified an acceptable level of consistency for this band (>0.7). Krause et al. [15] subsequently described a poor reliability between measures in an auditory memory task for the alpha band. In a more recent study, Neuper [16] indicated that the frequency and brain region were critical for the reliability in this technique. Moreover, Friedrich et al. [17] conducted a study in which they considered not only brain region and frequency band, as suggested by Neuper, but also the task type as another variable. In their results, the temporal stability was variable between tasks and frequencies over brain regions and time intervals. Cronbach’s alpha coefficients range from 0.07 to 0.90, which indicates that there is a heterogeneous behaviour for this band in different cognitive tasks, as proposed by the authors. In the seven tasks analysed in this study, only one task scored 0.07, and the remaining tasks were all greater than 0.77.

As presented in a previous report from our group [18], test-retest studies have typically been performed at a group level. However, an interesting analysis for the potential clinical applications of this alpha ERD is to determine whether the individual maps for each subject exhibit a high correlation coefficient between measures. This could enable a longitudinal follow-up for each subject in cases in which a therapeutic strategy is applied (e.g., pharmacological treatment or neuropsychological rehabilitation) or a cognitive or clinical follow-up is needed.

To our knowledge, there are no previous studies regarding the individual reliability scores for the alpha band using ERD analysis. In particular, the latency and amplitude of the maximum “valley” after the onset of the target stimulus will be explored. Moreover, the specific topography at this latency will be computed for each subject as was performed in the study of the P3 component.

An interesting benefit in this study is that we used the same EEG data that were employed in a previous publication [18], which will enable a comparison of the reliability scores of P3 and ERD in the same experimental procedure. Moreover, the coefficient of variation will be calculated to determine the potential application of alpha ERD in the clinical field and compare it to P3 values.

### Predictions

The first prediction is that the latency of the ERD-alpha will be stable between sessions and the amplitude will be increased in the second session in a similar manner as the P3 component. The second prediction is that the reliability of the alpha topography at the group level will be high when a visual oddball paradigm is used. This would be caused by the simplicity of this cognitive task compared with other more complex cognitive setups (i.e., memory). The third prediction is that the individual alpha topography will be highly specific for each subject between two sessions. However, the correlation scores will be in a range of moderate to high considering a higher heterogeneity in the alpha modulations than the P3 component. Finally, the heterogeneity in the alpha topography will exhibit a complex pattern in the comparison between subjects, which may represent different profiles in the ERD modulations for human subjects.

## Materials and methods

### Ethics statement

This study was conducted in compliance with the Helsinki Declaration. All participants signed informed consents prior to their inclusion, and the protocol was approved by the ethics committee of the University of Seville (project code: PSI2016-78133-P).

### Participants

The present sample was used in a previous study [18]. Thirty adults were recruited from university students and faculty staff. No subjects had a significant neurological history or drug consumption. EEG data were acquired in two EEG sessions separated by an average period of 48.5 ± 47.1 days (with a range from one week to three months). After review of the EEG traces, eight subjects were rejected because of the impossibility of removing the artefacts in at least one electrode of the 58 montage.

No interpolation procedures that could affect the topographical analysis were applied.

The final sample consisted of 22 adults (gender: 8 males, 14 females) (age: 21 to 50 years; mean 28.3 ± 7.68) (handedness: only one subject was left-handed).

### Cognitive task

The cognitive task employed was a “visual oddball”, in which the subject had to discriminate uncommon visual stimuli in a sequence of frequent stimuli. The target stimulus consisted of a rectangle with a checkerboard pattern that comprised red and white squares (appearance probability: 25%). The standard (frequent) stimulus was equivalent in size with the same pattern but with black and white squares. Both stimuli were displayed alternatively in the centre of the screen. A fixation point was present when no stimuli were presented to avoid changes in eye position during the experiment. The screen was located 70 centimetres from the participant’s eyes, and the size of both stimuli was 7.98 visual angle on the X axis and 9.42 on the Y axis. All stimuli were presented for 500 milliseconds (ms), and the interstimulus interval was one second, during which the subject could respond. One block with 200 trials was used in a pseudorandom presentation. The task for the participants was to press the mouse button with the right index finger when a target stimulus appeared and ignore the standard stimulus. At the end of the experimental session, the reaction time and percentage accuracy (for the target and overall, including no responses for the standard stimuli) were calculated.

### EEG procedure

**Fig 1:**
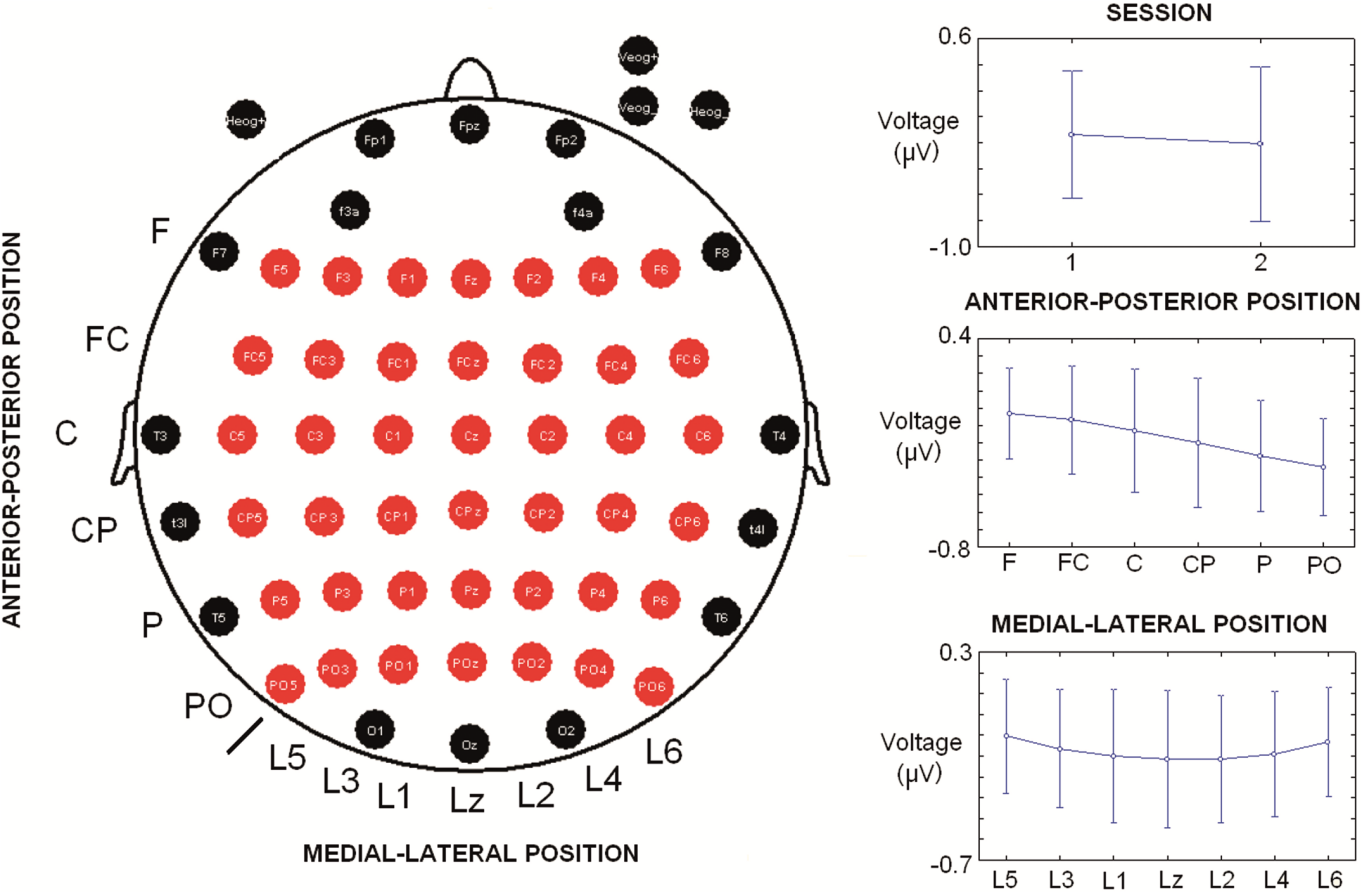
Electrode array and statistically significant factors in the ANOVA of the amplitude. The left side indicates 58 scalp electrodes and the ones used for the analysis in the present study (red). On the right side, graphics for main factors that were statistically significant in the ANOVA are displayed. Abbreviations: F (frontal), FC (Frontocentral), C (Central), CP (Centroparietal), P (Parietal), PO (Parietooccipital), L (line), z (zero or midline).

The electroencephalogram was recorded from 58 scalp electrodes (Fp1, Fpz, Fp2, AF3, AF4, F7, F5, F3, F1, Fz, F2, F4, F6, F8, FC5, FC3, FC1, FCz, FC2, FC4, FC6, T3, C5, C3, C1, Cz, C2, C4, C6, T4, TP7, CP5, CP3, CP1, CPz, CP2, CP4, CP6, TP8, T5, P5, P3, P1, Pz, P2, P4, P6, T6, PO7, PO3, PO1, POz, PO2, PO4, PO8, O1, Oz, and O2; refer to Fig 1 for detailed locations of recording derivations). All electrodes were referenced during the recording to the linked earlobe channel and offline rereferenced to an averaged reference. The ground electrode was placed in the mid-forehead. Vertical and horizontal electrooculograms (VEOG and HEOG, respectively) were recorded with bipolar recordings from electrodes situated in the inferior and superior positions of the left orbit and the external canthi of the ocular orbits, respectively. The electrode signals were amplified using BrainAmp amplifiers (Brain Products GmbH, Gilching, Germany) and were digitally stored using Brain Vision Recorder software (Brain Products GmbH, Gilching, Germany). The EEG signal was digitized at a frequency of 500 Hz and filtered in the amplifier using a bandpass of 0.01–100 Hz with the impedance less than 5 kOhm during the experiment. The following protocol was applied to calculate the event-related desynchronization (ERD): ocular correction of the blinking artefact in the scalp electrodes using the algorithm developed by Gratton et al., [19]; segmentation of the continuous EEG recording (-100 to 1000 ms, with zero indicating the onset of the target stimulus); baseline correction based on the previous interval to the stimulus (-100 to 0 ms); visual review of EEG epochs and rejection of artefacts. Moreover, the trials in which the HEOG signal was outside the ±75 μV range were rejected. A subsequent bandpass filtering was included in the desired band (10–14 Hz), which rectified the signal and was ultimately averaged for the target condition and subject. As recommended by Polich [20], all individual averages comprised a similar number of artefact-free trials to avoid misinterpretations as a result of different signal-to-noise ratios. In the present study, sessions 1 and 2 comprised 47.9 and 48.4 trials, respectively. There was no significant difference in the number of trials between sessions, t = -0.68, p = 0.498.

The latency and amplitude values of the alpha ERD were calculated in the electrode that showed the maximum amplitude for each subject. The alpha “valley” was identified as the maximum negativity in the interval between 250 and 700 milliseconds. A low-pass filter (30 Hz (48 dB/octave)) was used to eliminate small high-frequency fluctuations and simplify the identification of the highest negative value of the alpha ERD [7]. Following the guidelines for the analysis of EEG parameters [21], the amplitude values for the remaining electrodes were exported in the latency for the topographical study.

The statistical analysis was initiated by checking all variables for normality using the Shapiro-Wilk test. To calculate potential differences among the sessions in the behavioural responses and the latency and amplitude of the alpha ERD, paired t-test for dependent variables was used.

For the study of topographical differences in the amplitude of the alpha ERD between the two sessions, an analysis of variance (ANOVA) was applied with the following factors: Factor 1: “Session” (levels (2): 1 and 2); Factor 2: “Anterior-posterior Position” of the electrode (levels (6): Frontal; Frontocentral; Central; Centroparietal; Parietal; and Parietooccipital); and Factor 3: “Lateral-Medial Position” (levels (7): from lateral left to lateral right, example: Line 5, Line 3, Line 1, Midline or Line zero (z), Line 2, Line 4, Line 6) (i.e., F5, F3, F1, Fz, F2, F4, F6) (refer to Fig 1 for the locations of the electrodes analysed). A Greenhouse-Geisser correction for sphericity was applied, and a Bonferroni correction was included in the post hoc analysis. In all cases, a probability of p < 0.05 was considered significant.

To analyse the correlations between the amplitudes of the alpha ERD in two sessions and within-subjects, the intra-class correlation test (ICC) was used. Pearson’s product-moment r was employed for the between-subject comparisons. The 0.05 significance level was divided by the number of contrasts made for both correlation analyses (within- and between-subjects) [22]. For the within-subject comparison, the new level of significance obtained was p = 0.002 (0.05 divided by 22 comparisons); for the between-subject correlation, the p value was established as <0.00001 (0.05/462 comparisons). The coefficient of variation (CV) was calculated for the EEG and behavioural parameters using the formula described by other authors [23] (Coefficient of Variation = (Standard Deviation/Mean) x 100).

## Results

### Behavioural data

None of the behavioural variables considered in the present study exhibited significant differences between sessions (reaction time: t = 1.10, p = 0.280; accuracy for the target stimulus: t = 0.641, p = 0.528; or accuracy of global performance: t = 0.302, p = 0.765) (refer to Table 1 for mean values). Regarding the individual analysis of the reaction time values, some subjects increased (29 ms) or decreased (24 ms) their values in the second session. With respect to the accuracy variable (target or global scores), the changes between sessions were minimal.

**Table 1:** Behavioural and alpha ERD parameters for each subject.

Subject	RT S1	RT S2	RT Dif	ACC T S1	ACC T S2	ACC T Dif	ACC G S1	ACC G S2	ACC G Dif	Lat S1	Lat S2	LatDif	Amp S1	Amp S2	AmpDif	Elect S1	Elect S2
1	408	406	2	94.5	94.5	0	80	78	2	296	290	-6	-0.61	-0.85	-0.24	Cb2	Cb2
2	279	279	0	100	100	0	100	100	0	328	450	122	-0.72	-0.62	0.1	P1p	P2p
3	308	284	24	100	100	0	100	100	0	320	394	74	-0.36	-0.38	-0.02	Cb2	P1p
4	324	315	9	99	99.5	-0.5	96	100	-4	552	540	-12	-0.62	-1.11	-0.49	Cb2	Cb2
5	343	337	6	98	98	0	92	96	-4	474	430	-44	-0.43	-0.57	-0.14	P5	P5
6	302	287	15	99.5	100	-0.5	98	100	-2	392	486	94	-0.59	-0.41	0.18	Oz	P4p
7	314	324	-10	99.5	100	-0.5	98	100	-2	414	368	-46	-0.51	-0.57	-0.06	Pz	P1p
8	318	294	24	100	100	0	100	100	0	506	496	-10	-0.31	-0.32	-0.01	Pzp	P2p
9	351	372	-21	99	98	1	98	92	6	354	328	-26	-0.2	-0.32	-0.12	O1	Cb2
10	270	253	17	100	99.5	0.5	100	100	0	454	454	0	-0.44	-0.3	0.14	Cb2	Pzp
11	295	324	-29	100	100	0	100	100	0	468	652	184	-0.3	-0.47	-0.17	Pzp	P1p
12	284	285	-1	100	99.5	0.5	100	98	2	426	498	72	-0.58	-1.11	-0.53	Pz	P1
13	333	318	15	100	99	1	100	96	4	388	378	-10	-0.65	-0.78	-0.13	P5	P1p
14	296	308	-12	99.5	100	-0.5	100	100	0	430	382	-48	-0.71	-0.63	0.08	P5	P2p
15	310	325	-15	100	99	1	100	96	4	412	570	158	-0.4	-0.41	-0.01	Pz	Pz
16	315	307	8	100	99.5	0.5	100	98	2	528	346	-182	-0.52	-0.31	0.21	Pz	P4p
17	342	326	16	98.5	99	-0.5	94	96	-2	384	374	-10	-0.24	-0.05	0.19	Oz	P2p
18	259	262	-3	100	100	0	100	100	0	502	490	-12	-0.61	-0.71	-0.1	P2p	O1
19	300	319	-19	99	98	1	98	96	2	364	432	68	-0.65	-1.2	-0.55	O1	P1p
20	300	293	7	99.5	100	-0.5	100	100	0	422	530	108	-0.34	-0.41	-0.07	P4	Cb1
21	351	326	25	98	99.5	-1.5	92	98	-6	500	358	-142	-0.23	-0.32	-0.09	P3	Cb1
22	312	316	-4	99.5	98.5	1	98	96	2	360	392	32	-0.54	-1.04	-0.5	Cb2	Cb2
**Mean**	**314**	**312**		**99.2**	**99.1**		**97.5**	**97.3**		**421**	**438**	**16.55**	**-0.48**	**-0.59**	**-0.11**		
**StdDev**	**32**	**34**		**1.2**	**1.2**		**4.5**	**4.7**		**70.44**	**88.16**	**88.76**	**0.16**	**0.31**	**0.23**		
**CV**	**10.3**	**10.8**		**1.3**	**1.3**		**4.8**	**5.0**		**16.71**	**20.12**		**33.33**	**52.54**			

Abbreviations. RT: Reaction time (in milliseconds). S1: Session 1. S2: Session 2. ACC: Accuracy. T: Target. G. Global (Target and Standard). Dif. Difference (S1 –S2). Lat. Latency (in milliseconds). Amp. Amplitude (in microvolts). Elect (electrode with the maximum amplitude value for ERD-alpha). CV: Coefficient of Variation.

With respect to the correlation analyses, all variables exhibited good values between sessions (RT r = 0.880, p < 0.001; global accuracy r = 0.860, p < 0.001; and target accuracy r = 0.827, p < 0.001).

### Alpha ERD (latency and amplitude)

The latency values for ERD did not exhibit significant intersession differences (session 1: 422 ± 70 ms; session 2: 438 ± 88 ms) (t = -0.874, p = 0.391) and exhibited a poor correlation score (r: 0.391 p = 0.072).

**Fig 2:**
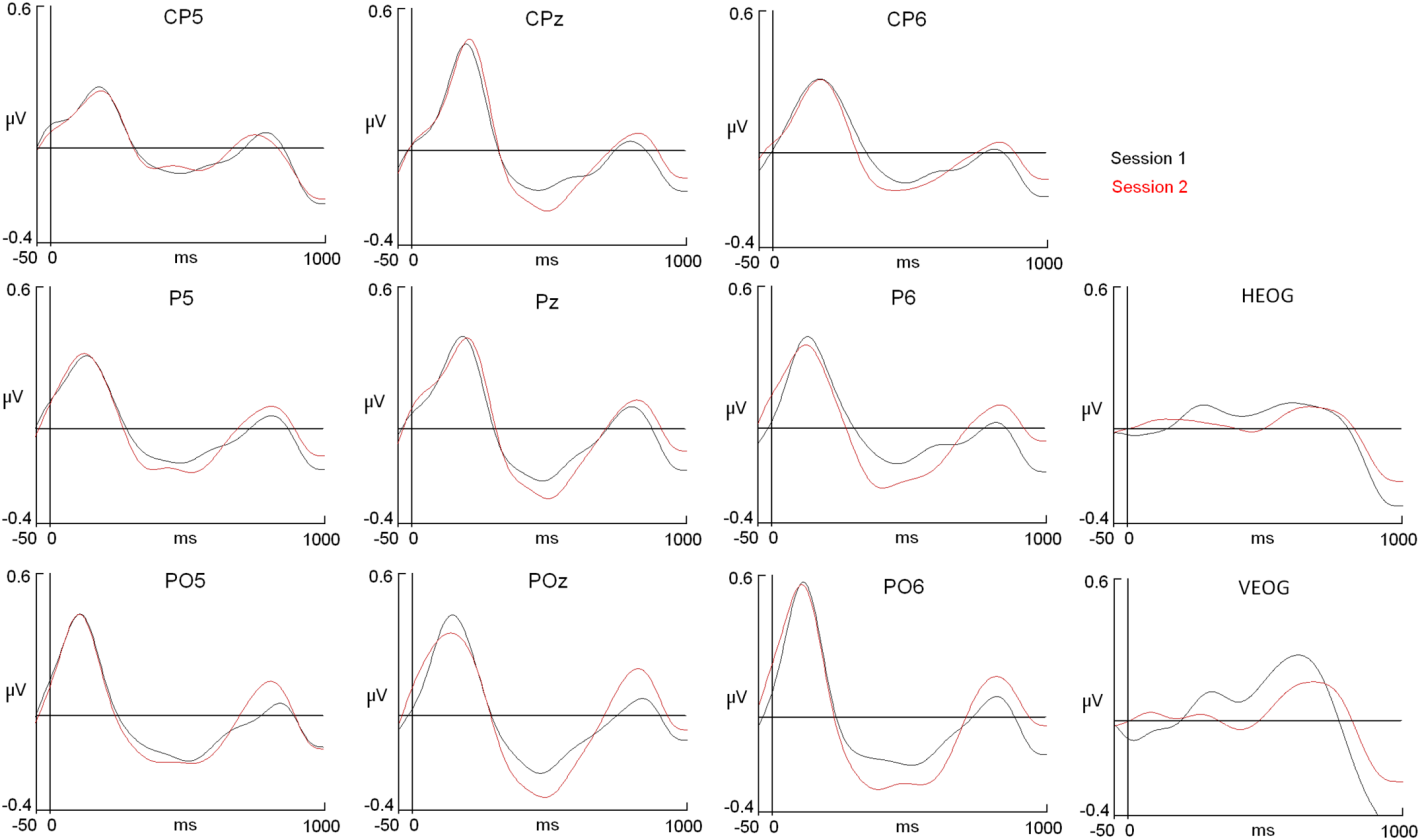
Alpha ERD modulation among sessions in visual oddball paradigm. The X axis represents “time” expressed in milliseconds (ms), and the Y axis represents the “amplitude” of the ERP in microvolts (μV). The onset of the stimulus is represented by the vertical dashed line. HEOG: Horizontal electrooculogram. VEOG: Vertical electrooculogram. Note the increase in the alpha ERD amplitude, particularly in posterior derivations.

Regarding amplitude, a statistically significant increase was identified in the second session (session 1: -0.48 ± 0.16 μV; session 2: -0.58 ± 0.31 μV) (t = 2.132, p = 0.044) (Table 1 and Fig 2). The correlation between the two sessions was higher than that obtained for the latency (r: 0.691, p < 0.001).

The alpha latency exhibited an average difference between measures of 16.5 ms; however, this low value and a low correlation score (0.391) was justified by considerable changes between sessions (in some cases, an increase of 184 ms or a decrease of 182 ms). In a previous study with the P3 component, changes less than 10 ms were identified in half of the sample. In the alpha latency, this result was identified in only 4 subjects. The amplitude showed a change of 0.55 microvolts in a particular case, and an increment (more negativity) was identified between sessions in 17 subjects.

Considering previous studies have referred the relevant influence of the alpha prestimulus in the subsequent behaviour of the alpha ERD [24], a specific analysis was performed in the previous 400 ms to the onset of the visual stimuli. The ANOVA factors for the alpha prestimulus were identical to those used for the alpha ERD, although in this case for the entire period (-400 to 0 ms, with zero indicating the onset of the target stimulus). The results indicated there were no differences between sessions for any factor involved in the analysis.

Another potential contribution may originate from eye artefacts; thus, we compared the mean values between sessions of the alpha ERD in ocular electrodes (HEOG and VEOG) for the interval (250 to 700 ms), and no significant differences were identified between sessions.

Finally, CV for all behavioural variables and alpha ERD parameters showed a heterogeneous set of values (Table 1). The lowest value corresponded to the accuracy for target performance (CV session 1: 1.3; session 2: 1.3). For the alpha parameters, the latency showed higher coefficients for both sessions (session 1: 16.7; session 2: 20.1) than P3 ((session 1: 8.3; session 2: 6.8) data from a previous study [18]); however, it remained less than 30, which is an acceptable value in some clinical variables [23]. In the case of amplitude, despite obtaining a better correlation score than the latency, the coefficients of variation were greater than 30, which indicates that this parameter is the most volatile parameter, particularly in session 2 (session 1: 33.3; session 2: 52.5).

### Alpha ERD topography (group analysis)

The ANOVA indicated three different main effects for the amplitude variable (Session: F(1,21) = 10.03, p = 0.004, η^2^: 0.323; Anterior-posterior Location: F(5,105) = 66.97, p<0.001, η^2^: 0.761; and Lateral-Medial Location F(6,126) = 7.65, p<0.001, η^2^: 0.267). Post hoc analysis showed that the “Session” main effect was explained by a general increment in the second session (session 1: -0.13; session 2: -0.21). The “Anterior-Posterior Location” main effect was a result of a higher amplitude of negativity in the posterior electrodes, which ranged from -0.03 microvolts in the frontal regions to -0.34 microvolts in the parieto-occipital derivations. Finally, the “Lateral-Medial location” main effect was determined by a higher negativity in the midline electrodes than the lateral positions in both sides of the scalp. The grand average maps (from different sessions) showed an excellent correlation score (r = 0.903, p<0.001).

### Alpha ERD topography (individual analysis, within-subjects)

As indicated in a previous study by our group for P3 [18], heterogeneity is present in the electrode that presented the maximum voltage for the alpha modulation in both sessions and for every subject (Table 1). In some cases, the change in the location was small (Pzp to P2p); however, in some subjects, the changes were more remarkable (from one side of the scalp to a contralateral position, i.e., O1 to Cb2).

When the intra-class correlations in topography for each individual participant were analysed, the results showed that 14 subjects were greater than 0.7, which is considered an acceptable [25] to excellent score (ranged from 0.717 to 0.954). Five of 22 subjects were questionable (ranged from 0.604 to 0.692), and 3 subjects showed poor correlation scores (ranged from 0.391 to 0.469) (refer to S1 Table for all values).

### Alpha ERD topography (individual analysis, between-subjects)

The between-subject comparison indicated that only nine subjects exhibited a higher within-subject correlation than between-subject correlations in session 1 (refer to S1 Table for specific values of all comparisons). Moreover, in session 2, nine subjects were more highly correlated with each other than they were with the remaining subjects; however, they were not the same subjects as in session 1.

Different profiles may be described comparing within-subject and between-subject correlation scores. Subject number 1, for example, exhibited a highly specific intra-class correlation (sessions 1 and 2), and it was always higher than any comparison with other subjects of the sample (in sessions 1 or 2).

Another profile is the one shown by subject number 9, in which a high intra-class correlation was identified for its topography (0.902), which was always higher than any comparison with other subjects in session 1. However, in session 2, subject 9 showed an even higher correlation with subject 22 (0.940). In contrast, subject number 11 displayed a high intra-class correlation score (0.903), whereas in the first session, two subjects showed a higher correlation with him (subject #2, 0.910; subject #8, 0.930).

A third type of profile is the one presented by subject #14, who exhibited a moderate intra-class score (0.717) but no subject showed a higher between-subject correlation with him. However, in session 2, some subjects obtained a higher correlation with him. Moreover, the opposite possibility was also identified in subject number #12, who presented higher correlation scores with some subjects in session 1 (#7 and 15), whereas in session 2, the intra-class score was higher than any between-subject comparison.

Finally, a remarkable result is that the number of between-subject comparisons that were higher than intra-class scores in session 2 (76) was two-fold with respect to session 1 (38).

## Discussion

### Behavioural data

As indicated in our previous study [18], behavioural responses showed a high reliability value at the group level, as described in other studies [26]. Reviewing the individual data, some subjects exhibited similar values between sessions, whereas other subjects reached more than 20 ms of difference. Despite this heterogeneity, the coefficients of variation were low (CV less than 30) [23], which thus supports the usefulness of these measures in the clinical environment.

### Alpha ERD latency and amplitude

The statistical analyses indicated that the latency does not change between sessions at the group level. It is important to consider that this result has been obtained in a visual oddball task, as other studies have indicated the importance of the task on reliability scores [17].

Furthermore, the amplitude showed an increase for session 2 as occurred with P3 in the same sample of subjects [18]. This alpha ERD increment may be interpreted as a better reduction of alpha activity to improve the performance of the subject, as proposed by other authors [27]. In our previous study, the P3 component exhibited a higher amplitude in the second session, which was interpreted as less resources involved in the information processing [18]. In the case of the alpha ERD, a steeper reduction of its amplitude may represent a more efficient recruitment of the neural processes involved in the alpha ERD modulation (which may represent less resources needed), which is likely based on a practice effect.

The correlation analysis indicated that the latency showed a low score between sessions. Despite the lack of differences between sessions for the mean values of the latency in both sessions, this low correlation score may be interpreted as a variable behaviour for the latency between sessions.

In the case of amplitude, the correlation score was better (0.691) and close to the acceptable level. Some studies have indicated that the amplitude has a better correlation score than the latency in other components, such as P3 [28, 29]. This better score is likely a result of a common increase in the alpha ERD for session 2. Comparing these results to the same parameters in the P3 component, it is possible to observe a better correlation in the time-domain component (P3) (r = 0.880) [18] than in the spectral-domain component (alpha ERD).

The main conclusion is that in the alpha modulation, more psychological processes are included than in the P3 component, or these are more changeable in their manifestation as neural activity. Additional studies are required to understand the number of structures involved and their functional roles in the alpha ERD elicited in this particular cognitive task.

### Alpha ERD topography (group analysis)

An excellent correlation score was obtained for the alpha ERD topography between sessions (ICC = 0.903). It is remarkable that the topography of the alpha ERD showed this high correlation score even when the latencies for exporting the voltage amplitudes were different among sessions. This high score indicates that the reliability for alpha ERD maps may be excellent depending on the task employed. Other studies have identified lower values for the test-retest [15], and the reason for this finding is likely the requirement of memory processes that significantly change the neural activity between sessions.

Regarding the potential modulations in amplitude for specific electrodes, an ANOVA was performed to analyse the potential changes between sessions. The results indicated there was no interaction between the Session factor and the Location factors, which suggests that no complex reorganization in the topography was present. A general increase in the second session and a predominant topography in the posterior midline electrodes were identified (Fig 2). A subtle trend to the right side of the scalp is indicated for alpha ERD. Some studies have described a right lateralization of alpha ERD [30], whereas other studies have shown a bilateral expression of the alpha decrement [31]. In an auditory oddball [6], the topography exhibited an early modulation in the left side of the scalp, and it subsequently moved to the right side. In the present study, the alpha decrement was higher and sharper for midline locations; however, the presence of a second rebound was clear in the right side of the scalp.

With regard to the increase of alpha ERD in the second session, authors [15] have described an increase that corresponds to a lower effort in a retrieval memory task. Moreover, studies have indicated no changes in longitudinal studies in control subjects [32, 33]. It appears necessary to conduct further research to disentangle the specific topographic modulations of alpha ERD and their relationship with specific requirements of the cognitive task.

A relevant issue regarding the potential differences obtained in alpha ERD is related to the prestimulus alpha content [24]. In our results, no changes were identified in the alpha band previous to the onset of the stimuli. It must be considered that no specific analyses have been conducted for each subject to determine whether low correlation scores may be caused by individual changes in the alpha prestimulus. However, the alpha increment identified in the second session at the group level does not appear to depend on the prestimulus alpha content.

### Alpha ERD topography (individual analysis, within-subjects)

**Fig 3:**
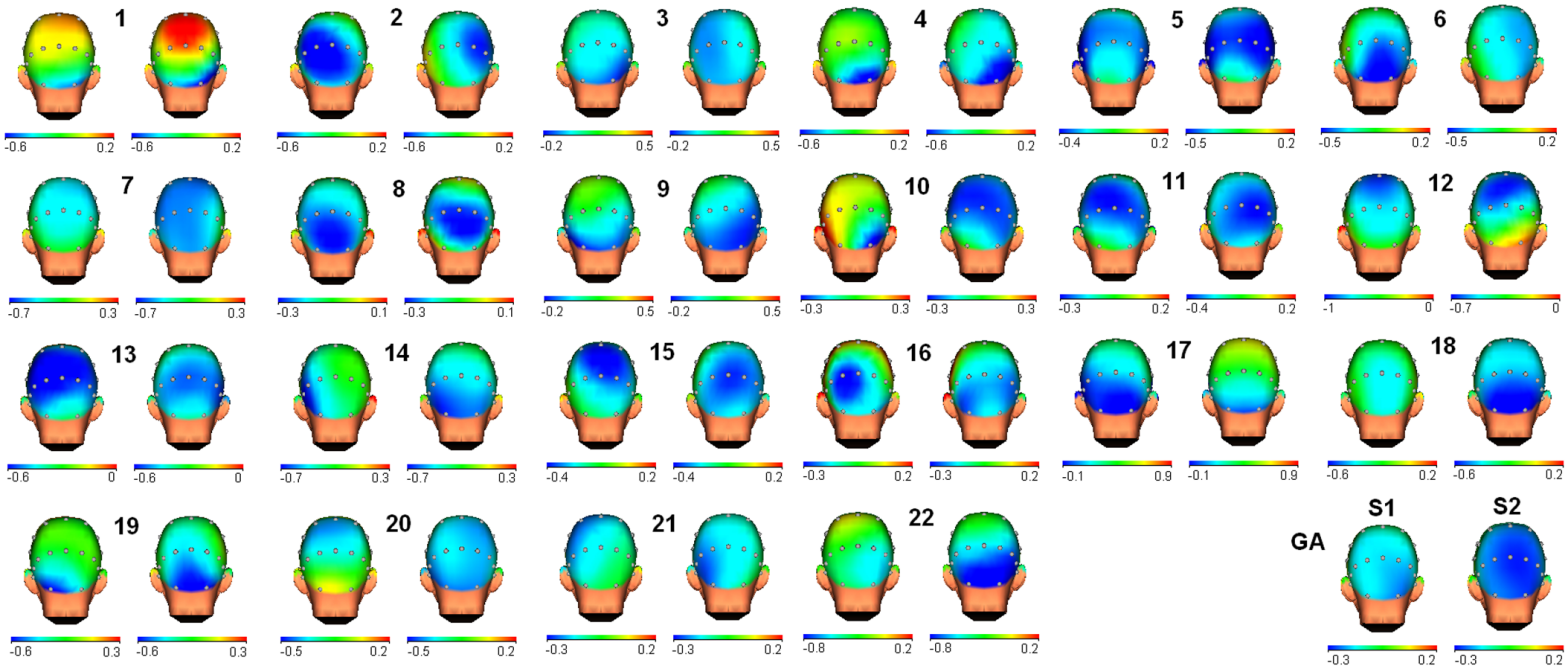
Topography for alpha ERD modulation in both sessions. Pairs of 3D head maps are shown for each of the 22 participants and the grand average (GA). The left side of the pair corresponds to session 1, and the right side corresponds to session 2. The scale (in microvolts) has been adjusted for each subject to clearly indicate the similarity or dissimilarity of the alpha ERD topography among sessions. Grand average maps exhibited an increase in this parameter in session 2.

When the within-subject comparison was conducted, the correlations between sessions were generally high (ICC mean: 0.750) (Fig 3). The highest and lowest correlation values were 0.954 and 0.391, respectively. Despite the acceptable mean value for the intra-class correlations, some subjects were below the category of acceptable (<0.7). Therefore, it appears that the individual maps for alpha ERD are not as stable as those obtained for the P3 component in the same sample and same cognitive task [18]. It is likely that the heterogeneity of the psychological processes involved in alpha ERD after the presentation of stimuli may reduce the stability between sessions. A plausible interpretation is that these maps were individually calculated when the grand averages could not camouflage specific modulations for each subject. To conclude, the grand average maps may be useful to detect changes in the topography of the alpha ERD; however, individual maps must be cautiously considered in the case of individual follow-up.

### Alpha ERD topography (individual analysis, between-subjects)

As indicated in the results section, different profiles were present when between-subject comparisons were made. A complex panorama was identified in which some subjects exhibited a highly specific topography and others were more similar to other subjects’ maps. Comparing these results with the P3 correlation scores for the same sample in a previous study [18], it is possible to conclude that alpha ERD is less reproducible than P3, which was clearly evident when multiple comparisons were conducted at the between-subject level. Moreover, this lack of reliability in the individual level is identified in a simple task, such as the visual oddball with one type of target and standard stimuli.

Contrasting the individual results, the group results evidence that common mechanisms in the alpha modulation are present in nearly all subjects. More homogeneity in the alpha ERD maps was identified not only in the first measure (session 1) but also in session 2, which may represent an automation or improvement in the processing that is present in the alpha increase after training. Previous studies have indicated an increase in alpha ERD related to Brain Computer Interface (BCI), rehabilitation [33] or induced recovery using transcranial Direct Current Stimulation (tDCS) [32].

### Clinical application

Regarding the potential application of alpha ERD in a clinical context, latency does not appear appropriate for it. It is promising that the alpha modulation is clearly distinguishable in the ERD modulation, and it was present in all subjects included in this study. However, some subjects showed that the modulation may present rebounds and multiple “valleys”, which complicates the decision regarding which point of measure is obtained. In this study, maximum negativity was selected to ensure that the experimenter did not bias the parameters of the alpha ERD modulation.

In the amplitude parameter, a general increase was identified that may be used to detect the lack of this increase in pathological groups. However, in the individual analysis, there was no global consistency in the entire sample (high coefficient of variation); therefore, its use is not recommended, at least for this cognitive task.

Topographical maps exhibit an excellent global reliability level, which suggests that the common mechanisms (group level) involved in the alpha ERD modulation may be very reliable between sessions. However, the individual maps are not globally consistent, which is likely a result of the heterogeneity in the alpha modulation identified in single subjects. Nevertheless, it is remarkable that topography is a robust characteristic considering that the difference in the latency when calculated for both sessions was greater than 100 milliseconds in some subjects (i.e., #11, 15 and 16).

In summary and considering the classification for correlation scores in psychological variables [25], nine subjects (40.1%) showed a good to excellent intra-class correlation score (> 0.8), five subjects (22.7%) were acceptable (0.6–0.7), five subjects (22.7%) were questionable (0.5–0.6) and three subjects (13.6%) exhibited a poor correlation score (0.3–0.5).

One remarkable result appears when the ICC scores of P3 and alpha ERD are compared (S1 Table). In general, the P3 ICC score is higher than that of the ICC for alpha ERD, as previously indicated. However, there are exceptions in which the alpha ERD showed excellent ICC scores higher than the individual P3 scores (subject #16, P3 ICC score = 0.902, alpha ERD ICC score: 0.915). This result suggests different mechanisms involved in alpha and P3 modulations (but not exclusive). Moreover, the reliability is higher in the P3 component; however, in some cases, subjects exhibit a higher reliability in the alpha ERD, which may depend on the cognitive setup that the subject has during the experiment. Clearly, further research to unveil all mechanisms involved in both components of the EEG signal is required to clarify these paradoxical results. Moreover, future studies should include a greater time lapse between sessions, which would enable the determination of whether group alpha ERD maps are sufficient parameters for longitudinal studies.

## Conclusion

In summary, different parameters of alpha ERD in a visual oddball task showed diverse behaviours in a test-retest reliability test. The latency did not significantly change between sessions; however, a low correlation score was obtained for this parameter. The amplitude increased in session 2 with respect to session 1 and presented a better correlation score than did the latency. The topography maps of alpha ERD were highly stable in the group analysis; however, they showed a wide range of intra-class correlation scores at the individual level. The between-subject comparisons revealed the presence of different profiles for alpha ERD modulation. A repetition of the task after 48 days (in average) caused an increase in alpha ERD, which could be related to automation and is represented as more homogeneous maps between subjects in the second session.

Compared with the P3 data obtained in a previous study with the same sample of subjects and cognitive task, alpha ERD appears less reliable than the P3 component, which could be due to a higher number of psychological processes involved or more changeable ones in the course of information processing.

## Supporting information

S1 Table Correlation scores for the alpha ERD topographical maps (within-subject and between-subjects). Within-subject correlations are represented by the ICC (Intra-class correlation, left column). The ICC scores for the P3 component from a previous study [18] are also presented. The correlation values for the topographical maps between subjects and both sessions were calculated by the Pearson product-moment. The values over the empty diagonal represent the correlations between subjects in session 1. The values for session 2 are shown below the empty diagonal. All values in bold are significant after Bonferroni correction (p<0.00001).
(DOC)
