# Supplementary material for: Retest reliability of individual alpha ERD topography assessed by human electroencephalography
Source: PLoS One. 2017 Oct 31;12(10):e0187244. doi: 10.1371/journal.pone.0187244 (PMC5663441; doi:10.1371/journal.pone.0187244)
Supplement: S1 Table — Within-subject correlations are represented by the ICC (Intra-class correlation, left column). The ICC scores for the P3 component from a previous study [18] are also presented. The correlation values for the topographical maps between subjects and both sessions were calculated by the Pearson product-moment. The values over the empty diagonal represent the correlations between subjects in session 1. The values for session 2 are shown below the empty diagonal. All values in bold are significant after Bonferroni correction (p<0.00001). (DOC) [file pone.0187244.s001.doc]

|  |  | **Subjects** |  |  |  |  |  |  |  |  |  |  |  |  |  |  |  |  |  |  |  |  |  |  |
| --- | --- | --- | --- | --- | --- | --- | --- | --- | --- | --- | --- | --- | --- | --- | --- | --- | --- | --- | --- | --- | --- | --- | --- | --- |
| **ICC** | **ICC P3** | **r Pearson** | 1 | 2 | 3 | 4 | 5 | 6 | 7 | 8 | 9 | 10 | 11 | 12 | 13 | 14 | 15 | 16 | 17 | 18 | 19 | 20 | 21 | 22 |
| 0,877 | 0.938 | 1 |  | **0,56** | **0,70** | **0,43** | **0,60** | **0,53** | -0,10 | **0,53** | **0,75** | **0,68** | **0,46** | 0,01 | **0,48** | 0,13 | -0,01 | 0,27 | **0,64** | **0,46** | 0,19 | -0,32 | **-0,51** | **0,73** |
| 0,675 | 0.981 | 2 | 0,30 |  | **0,63** | 0,27 | **0,76** | **0,56** | **0,46** | **0,91** | **0,75** | **0,54** | **0,91** | **0,40** | **0,90** | **0,52** | **0,50** | **0,63** | **0,71** | **0,82** | 0,35 | 0,13 | -0,01 | **0,68** |
| 0,725 | 0.934 | 3 | 0,18 | **0,50** |  | **0,71** | **0,41** | **0,53** | 0,35 | **0,54** | **0,64** | **0,78** | **0,56** | **0,55** | **0,60** | 0,22 | **0,58** | **0,68** | **0,49** | **0,58** | 0,30 | -0,01 | -0,39 | **-0,76** |
| 0,692 | 0.958 | 4 | **0,63** | **0,58** | **0,58** |  | 0,22 | **0,46** | **0,42** | 0,31 | **0,48** | **0,62** | 0,31 | **0,57** | 0,35 | -0,19 | **0,49** | **0,43** | 0,15 | 0,36 | 0,01 | 0,17 | **-0,41** | **0,54** |
| 0,898 | 0.766 | 5 | 0,25 | **0,53** | 0,39 | **0,49** |  | **0,46** | 0,38 | **0,80** | **0,76** | **0,42** | **0,78** | 0,23 | **0,83** | **0,48** | 0,31 | 0,36 | **0,52** | **0,64** | 0,36 | 0,25 | -0,12 | **-0,59** |
| 0,746 | 0.923 | 6 | 0,22 | **0,84** | **0,57** | **0,70** | **0,46** |  | **0,44** | **0,67** | **0,61** | **0,73** | **0,59** | 0,32 | **0,57** | -0,08 | **0,43** | **0,49** | 0,28 | **0,63** | 0,13 | -0,07 | **-0,56** | **0,72** |
| 0,687 | 0.968 | 7 | -0,14 | 0,27 | **0,65** | **0,47** | 0,37 | 0,37 |  | **0,56** | 0,31 | 0,32 | **0,61** | **0,83** | **0,63** | 0,12 | **0,83** | **0,58** | -0,12 | **0,52** | 0,13 | **0,53** | -0,11 | 0,37 |
| 0,673 | 0.797 | 8 | 0,24 | **0,51** | **0,82** | **0,59** | **0,44** | **0,52** | **0,66** |  | **0,75** | **0,57** | **0,93** | **0,41** | **0,89** | 0,35 | **0,52** | **0,54** | **0,54** | **0,88** | 0,21 | 0,21 | -0,24 | **0,68** |
| 0,902 | 0.701 | 9 | **0,65** | **0,74** | **0,42** | **0,67** | **0,72** | **0,57** | 0,19 | **0,56** |  | **0,65** | **0,74** | 0,28 | **0,75** | 0,32 | 0,27 | **0,46** | **0,66** | **0,65** | 0,32 | 0,13 | -0,38 | **0,83** |
| 0,751 | 0.626 | 10 | 0,28 | **0,74** | **0,79** | **0,64** | **0,68** | **0,66** | **0,61** | **0,76** | **0,72** |  | **0,52** | **0,43** | **0,50** | 0,01 | **0,42** | **0,55** | 0,37 | **0,57** | 0,25 | -0,16 | **-0,57** | **0,79** |
| 0,903 | 0.979 | 11 | 0,28 | **0,81** | **0,79** | **0,68** | **0,66** | **0,81** | **0,56** | **0,79** | **0,71** | **0,86** |  | **0,50** | **0,92** | **0,40** | **0,60** | **0,54** | **0,54** | **0,90** | 0,17 | 0,28 | -0,22 | **0,63** |
| 0,765 | 0.954 | 12 | -0,33 | **0,42** | **0,57** | 0,30 | **0,62** | **0,50** | **0,72** | **0,51** | 0,26 | **0,65** | **0,63** |  | **0,50** | **0**,12 | **0,84** | **0,63** | -0,08 | **0,46** | 0,14 | **0,52** | -0,14 | **0,41** |
| 0,604 | 0.892 | 13 | **0,40** | **0,65** | 0,31 | **0,54** | **0,43** | **0,57** | 0,10 | **0,41** | **0,66** | **0,46** | **0,53** | 0,15 |  | **0,49** | **0,61** | **0,58** | **0,53** | **0,81** | 0,37 | 0,31 | -0,11 | **0,64** |
| 0,717 | 0.945 | 14 | **0,54** | **0,48** | **0,69** | **0,65** | **0,62** | **0,40** | **0,42** | **0,81** | **0,77** | **0,77** | **0,75** | 0,38 | **0,49** |  | 0,21 | 0,23 | **0,47** | 0,30 | **0,62** | 0,18 | **0,50** | 0,17 |
| 0,867 | 0.942 | 15 | 0,23 | **0,79** | **0,65** | **0,64** | **0,61** | **0,75** | **0,46** | **0,72** | **0,72** | **0,75** | **0,85** | **0,58** | **0,61** | **0,66** |  | **0,72** | -0,02 | **0,55** | 0,17 | **0,47** | -0,09 | 0,37 |
| 0,915 | 0.902 | 16 | **0,33** | **0,77** | **0,57** | **0,58** | **0,42** | **0,80** | 0,27 | **0,56** | **0,60** | **0,61** | **0,81** | 0,38 | **0,54** | **0,53** | **0,84** |  | 0,27 | **0,44** | 0,23 | 0,15 | -0,25 | **0,62** |
| 0,862 | 0.865 | 17 | **0,61** | 0,13 | 0,31 | 0,37 | 0,11 | -0,01 | 0,04 | **0,59** | **0,50** | 0,27 | 0,29 | -0,25 | 0,29 | **0,71** | 0,26 | 0,20 |  | **0,49** | 0,30 | -0,06 | -0,04 | **0,56** |
| 0,918 | 0.977 | 18 | **0,52** | **0,79** | **0,64** | **0,76** | **0,74** | **0,67** | **0,45** | **0,73** | **0,89** | **0,88** | **0,83** | **0,50** | **0,64** | **0,84** | **0,82** | **0,68** | **0,44** |  | 0,26 | 0,25 | -0,22 | **0,56** |
| 0,469 | 0.865 | 19 | 0,27 | **0,45** | **0,70** | **0,44** | 0,15 | 0,36 | **0,49** | **0,76** | **0,46** | **0,59** | **0,61** | 0,28 | 0,28 | **0,60** | **0,68** | **0,55** | **0,51** | **0,59** |  | 0,06 | **0,40** | 0,29 |
| 0,391 | 0.889 | 20 | 0,21 | **0,53** | **0,40** | **0,42** | **0,83** | **0,43** | 0,39 | **0,56** | **0,71** | **0,69** | **0,68** | **0,62** | **0,42** | **0,75** | **0,59** | **0,42** | 0,31 | **0,78** | 0,28 |  | 0,30 | -0,04 |
| 0,482 | 0.683 | 21 | 0,06 | 0,08 | **0,50** | **0,40** | **0,47** | 0,14 | 0,60 | **0,67** | 0,35 | **0,48** | **0,43** | **0,54** | 0,23 | **0,68** | **0,56** | 0,32 | 0,39 | **0,50** | **0,50** | **0,55** |  | **-0,59** |
| 0,954 | 0.973 | 22 | **0,77** | **0,71** | **0,48** | **0,73** | **0,65** | **0,59** | 0,15 | **0,59** | **0,94** | **0,69** | **0,73** | 0,17 | **0,68** | **0,80** | **0,71** | **0,69** | **0,56** | **0,87** | **0,50** | **0,63** | 0,33 |  |
